# FPOP-LC-MS/MS Suggests Differences in Interaction Sites of Amphipols and Detergents with Outer Membrane Proteins

**DOI:** 10.1007/s13361-016-1421-1

**Published:** 2016-06-24

**Authors:** Thomas G. Watkinson, Antonio N. Calabrese, James R. Ault, Sheena E. Radford, Alison E. Ashcroft

**Affiliations:** Astbury Center for Structural Molecular Biology, School of Molecular and Cellular Biology, University of Leeds, Leeds, LS2 9JT UK

**Keywords:** Fast photochemical oxidation, Membrane proteins, Amphipols, Detergents, Structural proteomics

## Abstract

Amphipols are a class of novel surfactants that are capable of stabilizing the native state of membrane proteins. They have been shown to be highly effective, in some cases more so than detergent micelles, at maintaining the structural integrity of membrane proteins in solution, and have shown promise as vehicles for delivering native membrane proteins into the gas phase for structural interrogation. Here, we use fast photochemical oxidation of proteins (FPOP), which irreversibly labels the side chains of solvent-accessible residues with hydroxyl radicals generated by laser photolysis of hydrogen peroxide, to compare the solvent accessibility of the outer membrane protein OmpT when solubilized with the amphipol A8-35 or with n-dodecyl-β-maltoside (DDM) detergent micelles. Using quantitative mass spectrometry analyses, we show that fast photochemical oxidation reveals differences in the extent of solvent accessibility of residues between the A8-35 and DDM solubilized states, providing a rationale for the increased stability of membrane proteins solubilized with amphipol compared with detergent micelles, as a result of additional intermolecular contacts.

**Electronic supplementary material:**

The online version of this article (doi:10.1007/s13361-016-1421-1) contains supplementary material, which is available to authorized users.

**Graphical Abstract:**
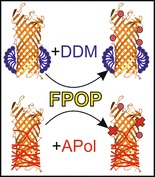
ᅟ

## Introduction

Despite the broad array of essential functions executed by membrane proteins (MPs), high resolution structural data for this class of proteins are lacking compared with their water-soluble counterparts. Electrospray ionization-mass spectrometry (ESI-MS) is emerging as an invaluable method with which to study MPs, allowing them to be transferred to the gas phase in a native-like state from a suitable amphiphile, and affording insights into MP mass, conformation, and small molecule binding [1–8]. n-Dodecyl-β-maltoside (DDM) is a frequently used detergent for such analyses [2–5] but recently the use of amphipols[1, 2, 8, 9] and other amphiphiles [6, 10] have been reported. Native MS can also be used in conjunction with other gas phase techniques, such as ion mobility spectrometry (IMS) [11–15] and collision induced dissociation and collision induced unfolding (CID/CIU) [16, 17] to probe the structure and topology of MPs and their complexes. A recent ESI-MS study of MP analyses using either DDM micelles or the polyacrylate-based amphipol A8-35 indicated that while both amphiphiles enabled transferral of a range of MPs into the gas phase, the proteins analyzed from amphipol retained a more native-like conformation, as judged from charge-state distributions and collision cross-sectional areas estimated from IMS measurements obtained within the same experiment [2]. These observations pose the question of whether there are differences in the ways that MPs interact with DDM and A8-35.

**Figure 1:**
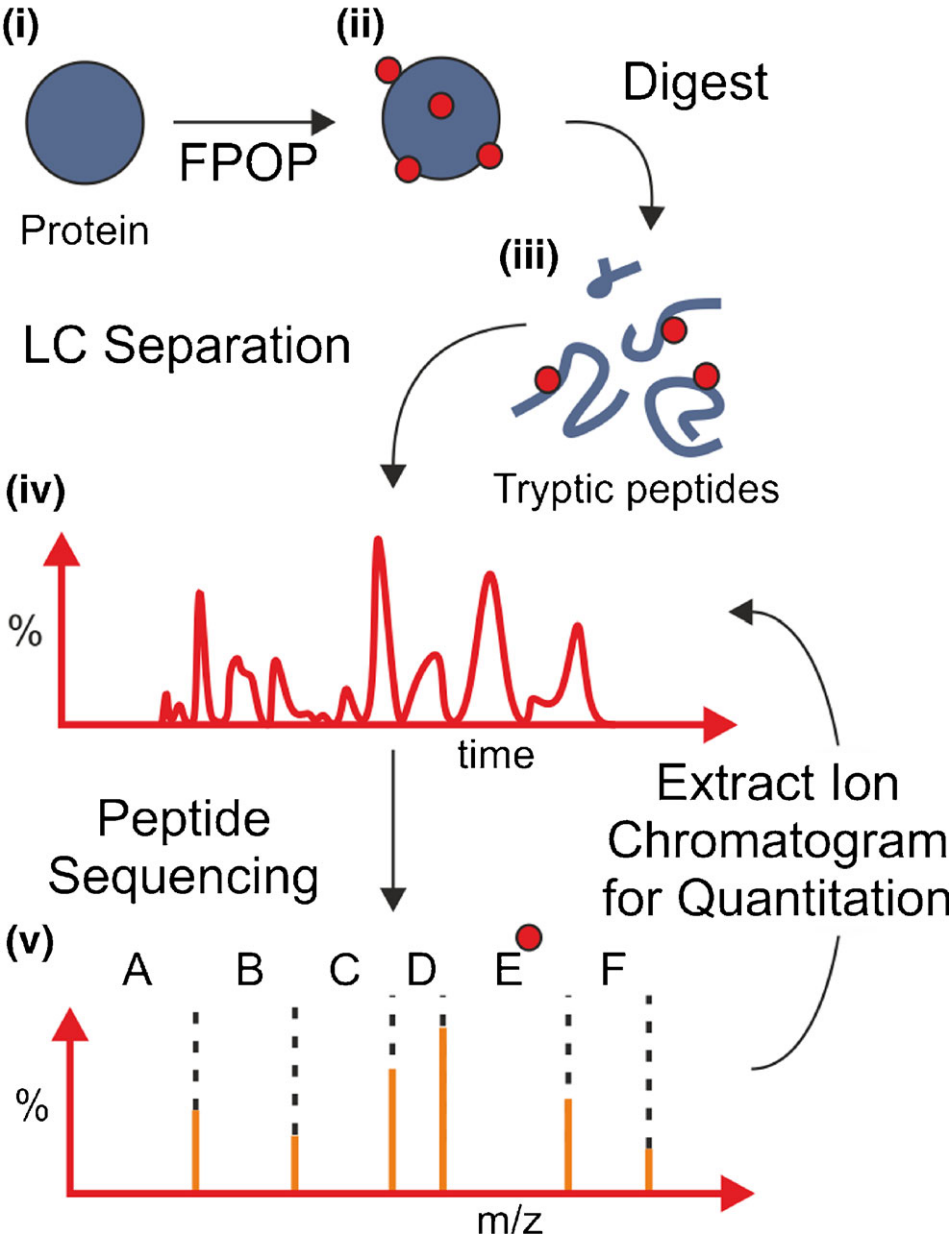
FPOP-MS work-flow. (i) Fast photochemical oxidation of proteins (FPOP) irreversibly labels solvent-accessible sites on protein side-chains; (ii) covalently modified proteins are digested to generate modified and unmodified tryptic peptides; (iii) LC-MS separation of these peptides is followed by (iv) MS/MS sequencing from which (v) a sequence can be derived for each peptide for identification of residue-specific modification sites. Modification sites are indicated by red circles

In the field of structural proteomics, MS methods are commonly employed following in-solution labeling techniques such as hydrogen deuterium exchange (HDX) [18–21], chemical cross-linking (XL) [22], or fast photochemical oxidation of proteins (FPOP) [23–27]. FPOP uses a KrF excimer laser to generate hydroxyl radicals from hydrogen peroxide, with which the protein is incubated. The radicals irreversibly oxidize the solvent-accessible side chains of the protein residues faster than most protein folding/unfolding events [24–27] (as a result of the hydroxyl radicals having an approximately 1 μs lifetime). The favorable attributes of FPOP include the fast labeling times and the irreversible nature of the chemical modifications, the latter permitting comprehensive downstream analysis using LC-MS/MS methods. Following such labeling experiments, the proteins can be subjected to proteolysis and the resulting peptides separated and sequenced using LC-MS/MS (Figure 1). Here, we apply FPOP-LC-MS/MS to gain insights into the interaction sites of two types of amphiphiles, the detergent DDM and the amphipol A8-35, with the 35.3 kDa β-barrel outer membrane protein OmpT. This protein comprises 10 β-strands, with both intermembrane and extramembrane regions, and functions in vivo as an endopeptidase [2, 8, 28, 29]. MP:amphipol complexes have been shown to be more stable over time than their detergent solubilized counterparts; whereas MPs solubilized in detergent micelles degrade over the range of hours to days, MP:amphipol complexes appear to remain stable indefinitely [9, 30, 31]. Here, the extent of protection from solvent afforded to OmpT by association with DDM detergent micelles or with the amphipol A8-35 has been evaluated using FPOP-LC-MS/MS. The results support the notion that additional contacts of amphipol with the extracellular regions of MPs can exist, which lead to a decrease in the solvent-accessible surface area of the MP, and which may play a key role in the increased stability afforded to MPs by amphipols.

## Methods

OmpT was overexpressed as inclusion bodies in *E. coli* BL21 (DE3) cells and purified by Ni^2+^-NTA affinity and size-exclsuion chromatography, as described previously [2, 8]. OmpT was then refolded into 0.5% (*w/v*) lauryldimethylamine-oxide detergent from its denatured state in 6 M guanidine.HCl and subsequently exchanged into 0.02% (*w/v*) DDM detergent [2, 8]. For analysis from A8-35, OmpT was trapped in the amphipol (Generon Ltd., Berkshire, UK) by adding A8-35 to DDM micelle-solubilized OmpT in a 1:5 (*w/w*) OmpT:amphipol ratio. After incubation for 1 h, the detergent was removed by overnight incubation with BioBeads (Bio-Rad, Hemel Hempstead, UK) at 4 °C, with gentle agitation.

For FPOP analysis, OmpT was buffer-exchanged into 10 mM sodium phosphate, 15 mM L-glutamine at pH 8.0 using Zeba Spin desalting columns (Thermo Fisher, Hemel Hempstead, UK) (supplemented with 0.02% (*w/v*) DDM for the detergent solubilized samples). Immediately before labeling, H_2_O_2_ was added to a final concentration of 0.05, 0.15, or 0.5% (*v/v*). The samples were infused through a fused silica capillary (i.d. 100 μm, with a window etched using a butane torch) at a flow rate of 20 μL/min through the path of a Compex 50 Pro KrF excimer laser operating at 248 nm (Coherent Inc., Ely, UK) with a pulse frequency of 15 Hz and a laser beam width of <3 mm at the point of irradiation. Hydroxyl radicals were generated by exposing H_2_O_2_ in the sample (through the etched window) to laser irradiation. These solution, flow, and laser pulse conditions ensure that each bolus of protein-containing solution is exposed only once to laser irradiation and that conformational averaging during labeling does not occur, as the labeling reaction is on a faster time scale than any unfolding event that may occur, due to the presence of the radical scavenger [24, 25]. The capillary outflow (100 μL) was collected in a 1.5 mL tube containing 20 μL of a 100 mM L-methionine/1 μM catalase solution in 10 mM sodium phosphate buffer, pH 7.0 to degrade any residual H_2_O_2_ and quench any hydroxyl radicals. Control samples were handled in the same fashion without being subjected to laser irradiation, to correct for any background oxidation that may occur on the timescale of the FPOP experiment.

After labeling, OmpT was digested by use of trypsin (1:20 (*w/w*) trypsin:OmpT ratio) at 37 °C for 24 h. The tryptic peptides were loaded onto a M-Class nanoAcquity LC system equipped with a C18 column, and analyzed using a Synapt G2Si (Waters Corp., Manchester, UK). MS^e^ data were acquired and processed using Waters’ UNIFI software. The degree of modification was measured as the % of total observed peptide that has been modified at one or more identified sites:

$$ \%\kern0.5em \mathrm{peptide}\kern0.5em \mathrm{modified}=\frac{{\displaystyle \sum Peak\kern0.5em  Are{a}_{modified}}}{{\displaystyle \sum Peak\kern0.5em  Are{a}_{modified}}+{\displaystyle \sum\ Peak\kern0.5em  Are{a}_{unmodified}\ }} $$

## Results and Discussion

**Figure 2:**
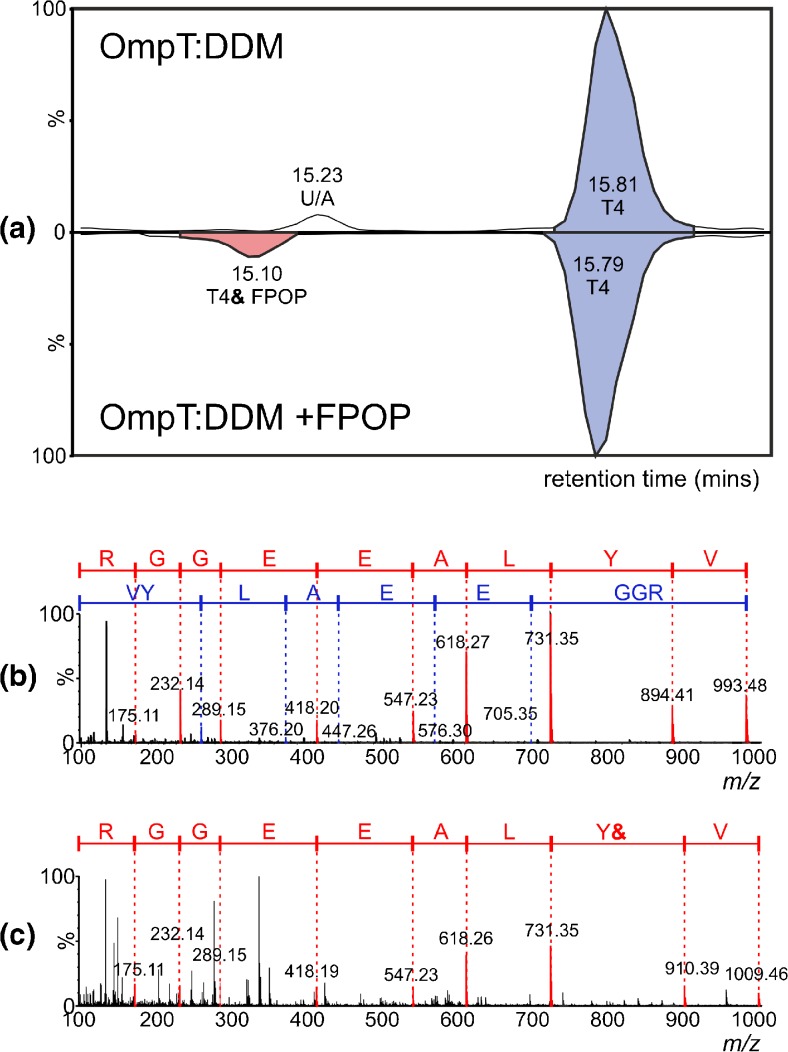
LC-MS/MS analysis of the OmpT tryptic peptide T4 (residues 43-51) following FPOP. (a) Butterfly plot of LC-MS chromatograms showing unmodified T4 (blue peaks) in the absence of (upper trace) and following (lower trace) FPOP, and oxidized T4 (pink peak, lower trace, labelled “T4& FPOP”) following FPOP. U/A indicates an unassigned peak that was not identified as a modified or unmodified OmpT tryptic peptide; (b) and (c) show MS/MS spectra of unmodified and oxidized T4, respectively. The y” ions are labeled in red and the b ions in blue; the location of the modification site (Y44) is shown by the “Y&” symbol

FPOP labeling of a protein can result in a number of covalent chemical modifications [24, 25, 32], with the most commonly encountered being the addition of an oxygen atom, accompanied by a mass increase of 16 Da. The change in protein mass can be monitored using ESI-MS, whilst the location of the modification can be identified at the amino acid residue level following proteolysis and MS/MS peptide sequencing (Figures 1 and 2).

Here, FPOP was used to compare the solvent accessible regions of OmpT in the presence of DDM detergent micelles or the amphipol A8-35. Control experiments were carried out to monitor and correct for the level of background oxidation (i.e., in the presence of H_2_O_2_ but absence of laser irradiation), which was found to be negligible in these experiments. Following trypsin digestion of modified OmpT, the resulting peptides were separated, identified, and sequenced using LC-MS/MS. As an example, Figure 2 shows data for the OmpT peptide T4 (the fourth tryptic peptide from the N-terminus of OmpT, residues 43-51, sequence VYLAEEGGR, mass 992.5 Da). It can be seen that FPOP oxidation of a protein can result in a small but reproducible decrease in the retention time of an oxidized tryptic peptide compared with its unmodified counterpart when using reverse-phase chromatography: Figure 2a shows the retention time window within which peptide T4 elutes (15-16 mins), comparing the control experiment (upper chromatogram) with the FPOP experiment (lower chromatogram). The retention times of unmodified peptide T4 (blue peaks in both chromatograms) and its modified counterpart (pink peak in lower chromatogram only) were found to be 15.8 and 15.1 min, respectively.

The MS/MS spectra for both unmodified and modified T4 peptides are shown in Figure 2b and c, with the y” (red font) and b (blue font) [33] ions highlighted. The 16 Da difference in mass between unmodified and oxidized T4 peptides is apparent (MH^+^ *m/z* 993.5 versus 1009.5) and the location of the modification was determined as the aromatic Tyr residue, Y44.

ESI-LC-MS/MS analysis of trypsin digested OmpT following FPOP from both DDM micelles and A8-35 yielded sequence coverages of 90%–95%. Of the 31 predicted tryptic peptides, 13 were found to be modified, with a total of 20 modification sites being identified in both amphiphiles (Supplementary Figure S1). The modified residues identified were either sulfur-containing or aromatics, as expected from the reported propensity of these groups to undergo oxidative labeling [32]. As expected from the relative reactivities of amino acid residues with free hydroxyl radicals, OmpT peptides containing Met residues (T8, T9, T12, T19) were modified to a greater degree than those labeled at Trp, Tyr, Phe, or His residues (T4, T6, T10, T11, T14, T15, T27, T30, and T31) (Supplementary Figure S1) [32]. Previous FPOP-MS studies of MPs reported only modifications at Met residues [20, 34]. In the comparative approach taken with this FPOP analysis, care must be taken to ensure that altering buffer conditions does not result in hydroxyl radical scavenging, which would lead to a general decreased extent of oxidation for all peptides. Here, the absence of a global trend (i.e., towards more or less oxidized) in either surfactant suggests minimal “scavenging” effects of DDM detergent micelles or A8-35 amphipol (Supplementary Figure S1); thus, the differences in oxidation observed here are most likely due to changes in solvent accessibility.

**Figure 3:**
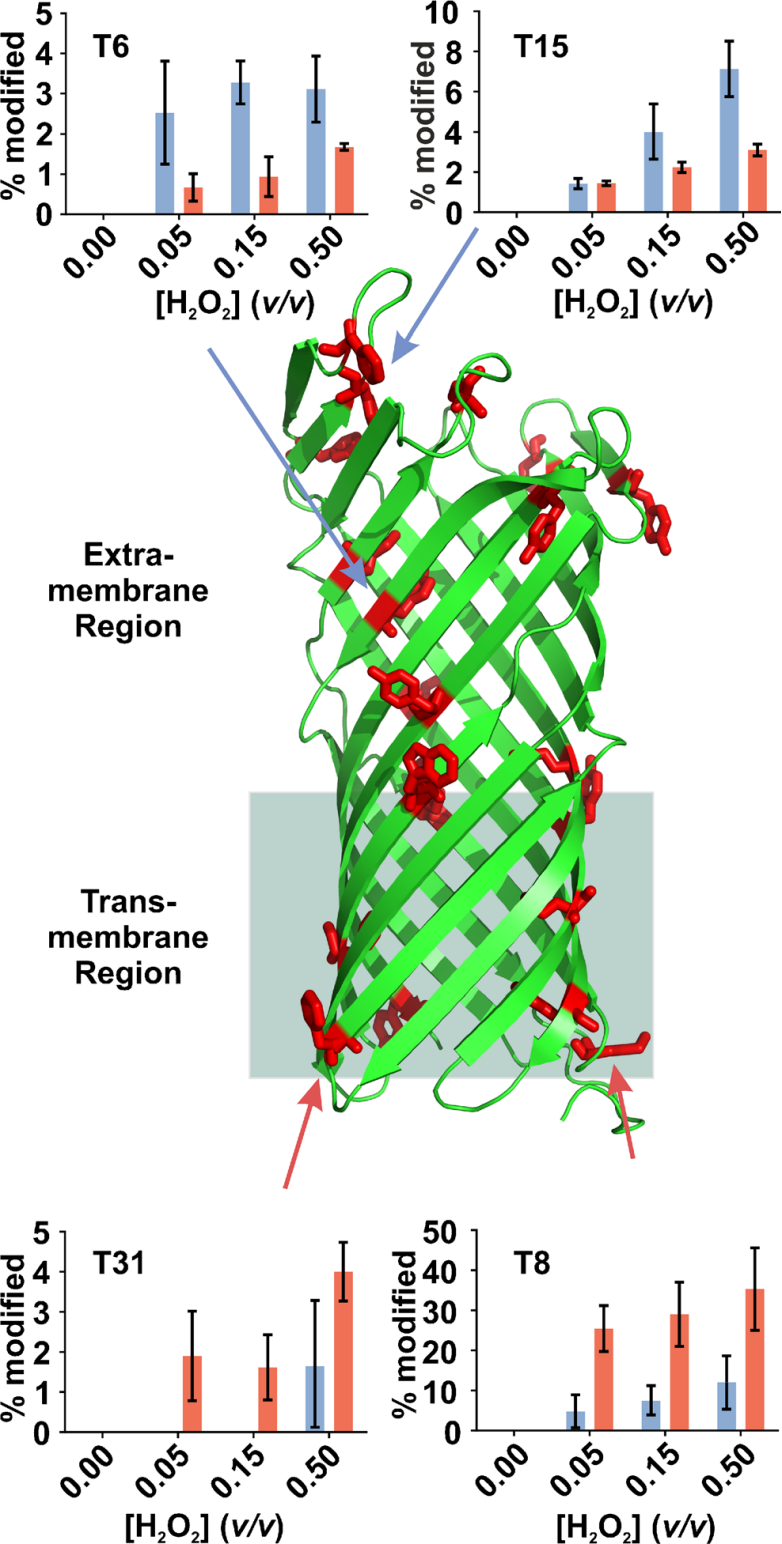
Tryptic peptides of OmpT (PDB 1I78 [29]) solubilized in DDM detergent micelles or in the amphipol A8-35 are modified to different degrees. Inset graphs show % peptide modified in DDM (blue) or in A8-35 (red) for four tryptic peptides of particular interest, and arrows (red and blue) indicate the respective residues that are modified in each peptide. Aromatic amino acid residues are shown in red. Residues towards the lower boundary of the transmembrane region are less readily labeled in DDM, whereas residues in the extra membrane region are shown to be less readily labeled in A8-35. Supplementary Figure S3 shows the location of the four tryptic peptides on the structure of OmpT

In vitro studies of MPs require an appropriate surfactant to mimic the native lipid bilayer in order to maintain their structural integrity. Although detergent micelles are the most commonly used surfactants, they have been noted to destabilize the native state of MPs [35, 36]. Also, it has been shown that OMPs exhibit a comparable global solution structure in both detergent and amphipol, although they differ in their solution-phase longevity [8]. Here, FPOP has been employed to identify differences in local structure in the presence of these two surfactants. Although the oxidation sites detected for OmpT were found to be the same regardless of the surfactant employed, the degree of modification of certain residues varied depending on whether the MP was solubilized in DDM detergent micelles or in the amphipol A8-35 (Supplementary Figure S1). Tryptic peptides with modification sites in the extra-membrane region of OmpT (e.g., T6 and T15) underwent more oxidation when solubilized in DDM than in A8-35 (Figure 3). FPOP of OmpT using 0.15% (v/v) H_2_O_2_, results in 3.3% of T6 and 4.0% of T15 being modified in DDM but only 0.9% of T6 and 2.2% of T15 undergoing modification in A8-35 (these data are the average of three replicates). Conversely, those peptides with modification sites in the lower boundary of the trans-membrane region (T8 and T31) were oxidized more when OmpT was solubilized in A8-35 than in DDM. FPOP using 0.15% (v/v) H_2_O_2_ resulted in 7.5% of T8 and 0% of T31 being modified in DDM, compared with 29.0% of T8 and 1.6% of T31 oxidation in A8-35.

We established previously that OmpT is natively folded (Supplementary Figure S2) and catalytically active in both DDM and A8-35 [1, 2], although MPs show significant differences in the thermal, chemical, and kinetic stability when solubilized in detergent micelles or amphipols [9, 30, 31]. Using ESI-IMS-MS, we have also shown that MPs analyzed from detergent micelles tend to occupy more expanded conformations, as indicated by the population of more highly charged ions with larger collision cross-sectional areas [2]. Regarding β-barrel OMPs, this phenomenon is greater for OmpT, with its large extra-membrane β-sheet region, than for PagP and tOmpA, both of which lack such a region [8]. Based on the results presented here, we propose that extra contacts of the amphipol A8-35 with this more exposed region of OmpT are likely to lead to a reduction in flexibility in the MP’s structure that prevents further charging during the ionization process (relative to DDM-solubilized protein).

A comparative NMR study of OmpX solubilized in the amphipol A8-35 or in di-hexanoyl-phosphocholine (DHPC) micelles showed that there was no obvious environment-dependent differences in the trans-membrane region of the protein [28, 37]. However, extra contacts of amphipols with MPs have been posited elsewhere: MD simulation structures of OmpX have shown A8-35 to interact with the extremes of the trans-membrane region, whereas detergent micelles do not [38], and NMR spectra of bacteriorhodopsin in DDM, compared with data obtained in the amphipol NAPol, display differences that have been attributed to contacts of NAPol with bacteriorhodopsin that are not present with DDM [39].

Differential interactions of A8-35 and DDM with OmpT can explain the differences observed in the data shown here. DDM micelles appear to associate better with the lower regions of the trans-membrane domain, protecting the M76 (peptide T8) and Y299 (peptide T31) residues from oxidative labeling. By contrast, A8-35 (either as free molecules in solution or as a polymer trailing from the trans-membrane region) can associate not only with the trans-membrane region of OmpT but also with its extra-membrane region, thus protecting residues such as W58 (peptide T6) and F177 (peptide T15) from oxidation.

## Conclusions

Here we have illustrated the utility of FPOP followed by ESI-LC-MS/MS to identify regional differences in the solvent accessibility of OmpT residues in different environments. The data highlight that detergent micelles and amphipol A8-35 interact with MPs differently, resulting in significant changes in solution phase properties.

## Electronic supplementary material

Below is the link to the electronic supplementary material.

