# Supplementary material for: FPOP-LC-MS/MS Suggests Differences in Interaction Sites of Amphipols and Detergents with Outer Membrane Proteins
Source: J Am Soc Mass Spectrom. 2016 Jun 24;28(1):50–5. doi: 10.1007/s13361-016-1421-1 (PMC5174144; doi:10.1007/s13361-016-1421-1)
Supplement: Supplementary file 1 [file 13361_2016_1421_MOESM1_ESM.docx]

FPOP-LC-MS/MS suggests differences in interaction sites of amphipols and detergents with outer membrane proteins

Thomas G. Watkinson, Antonio N. Calabrese, James R. Ault,

Sheena E. Radford*, Alison E. Ashcroft*

Astbury Centre for Structural Molecular Biology, School of Molecular and Cellular Biology, University of Leeds, Leeds, LS2 9JT, UK.

**Supporting Information**

| Tryptic  Peptide | Degree of modification (% total peptide with identified oxidation site) +/- SEM | | | | | | | | Location |
| --- | --- | --- | --- | --- | --- | --- | --- | --- | --- |
|  | n dodecyl-β-maltoside (DDM) | | | | amphipol A8-35 | | | |  |
|  | 0 % H_2_O_2_ | 0.05 % H_2_O_2_ | 0.15 % H_2_O_2_ | 0.5 % H_2_O_2_ | 0 % H_2_O_2_ | 0.05 % H_2_O_2_ | 0.15 % H_2_O_2_ | 0.5 % H_2_O_2_ |  |
| T4 | 0 | 1.95 +/- 0.10 | 2.72 +/- 0.10 | 2.96 +/- 0.05 | 0 | 1.32 +/- 0.14 | 2.36 +/- 0.2 | 2.80 +/- 0.06 | Y44 |
| T6 | 0 | 2.52 +/- 1.29 | 3.28 +/- 0.54 | 3.11 +/- 0.82 | 0 | 0.67 +/- 0.34 | 0.94 +/- 0.50 | 1.68 +/- 0.08 | W58 |
| T8 | 0 | 4.76 +/- 4.18 | 7.48 +/- 3.74 | 12.00 +/- 6.63 | 0 | 25.47 +/- 5.72 | 29.03 +/- 8.00 | 35.31 +/- 10.28 | M75,W85 |
| T9 | 0 | 69.93 +/- 9.4 | 80.39 +/- 10.4 | 92.08 +/- 4.83 | 0 | 42.70 +/- 10.32 | 54.67 +/- 7.58 | 81.02 +/- 3.35 | M95,M101 |
| T10 | 0 | 0.50 +/- 0.30 | 2.60 +/- 0.79 | 4.04 +/- 0.24 | 0 | 1.11 +/- 0.81 | 2.54 +/- 0.80 | 2.75 +/- 0.51 | Y122 |
| T11 | 0 | 2.43 +/- 0.51 | 5.05 +/- 0.90 | 6.02 +/- 0.68 | 0 | 2.11 +/- 0.31 | 4.15 +/- 0.48 | 5.52 +/- 0.14 | W133 |
| T12 | 0 | 14.99 +/- 0.83 | 18.71 +/- 0.73 | 17.64 +/- 7.53 | 0 | 14.92 +/- 1.88 | 19.85 +/- 2.91 | 30.22 +/- 2.67 | M145,Y148 |
| T14 | 0 | 2.97 +/- 1.12 | 6.47 +/- 0.95 | 9.96 +/- 0.84 | 0 | 3.60 +/- 1.50 | 6.28 +/- 1.13 | 8.47 +/- 0.75 | Y164,F170 |
| T15 | 0 | 1.42 +/- 0.26 | 4.01 +/- 1.37 | 7.13 +/- 1.39 | 0 | 1.44 +/- 0.12 | 2.24 +/- 0.26 | 3.10 +/- 0.29 | F177 |
| T19 | 0 | 9.72 +/- 4.93 | 19.36 +/- 0.46 | 23.52 +/- 1.53 | 0 | 16.33 +/-0.85 | 24.86 +/- 0.11 | 35.09 +/- 1.82 | M192 |
| T27 | 0 | 2.64 +/- 0.23 | 5.88 +/- 1.74 | 9.91 +/- 4.47 | 0 | 1.71 +/- 0.40 | 1.71 +/ -0.24 | 2.04 +/- 0.09 | Y262,W267 |
| T30 | 0 | 4.86 +/- 0.04 | 7.03 +/-1.00 | 6.98 +/- 0.91 | 0 | 5.20 +/- 0.96 | 6.77 +/- 1.25 | 5.80 +/- 0.67 | Y280,H282,Y289 |
| T31 | 0 | 0.00 | 0.00 | 1.64 +/-1.58 | 0 | 1.90 +/- 1.12 | 1.62 +/- 0.81 | 4.00 +/- 0.73 | Y299 |

Supplementary Figure 1. The degree of modification of OmpT peptides is reported as the % of total observed peptide that has one or more identified oxidation sites. OmpT is modified to varying extents at identified oxidation sites when OmpT is solubilised in DDM detergent or A8-35 amphipol and is labelled in 0, 0.05, 0.15 and 0.5 % H_2_O_2_ (*v/v*). Peptide nomenclature (TX) represents the peptide that precedes the X^th^ tryptic cut-site residue (K/R). Residues with multiple identified oxidation sites are more difficult to quantify at the residue level and more difficult to rationalise with spatially separated residues.


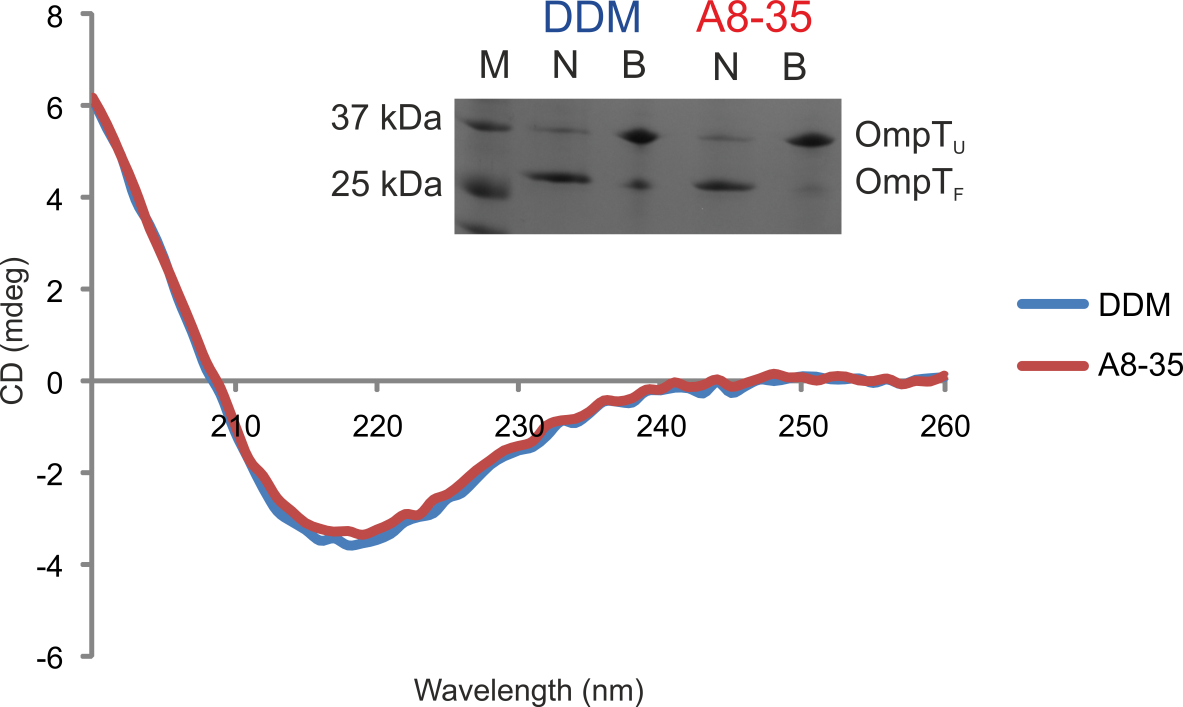


Supplementary Figure 2. Circular Dichroism (CD) and cold SDS-PAGE (inset) show OmpT to be in a compact, β-barrel structure in either DDM detergent (blue) or A8-35 Apol (red). Minima at 218 nm in the CD spectra are indicative of β-sheet secondary structure and presumably β-barrel topology. OmpT samples migrate at an apparent lower molecular weight (OmpT_F_) when loaded natively (N) and at their approximate molecular weight (OmpT_U_) when boiled prior to loading (B). (M) indicates the Marker lane on the SDS-PAGE.


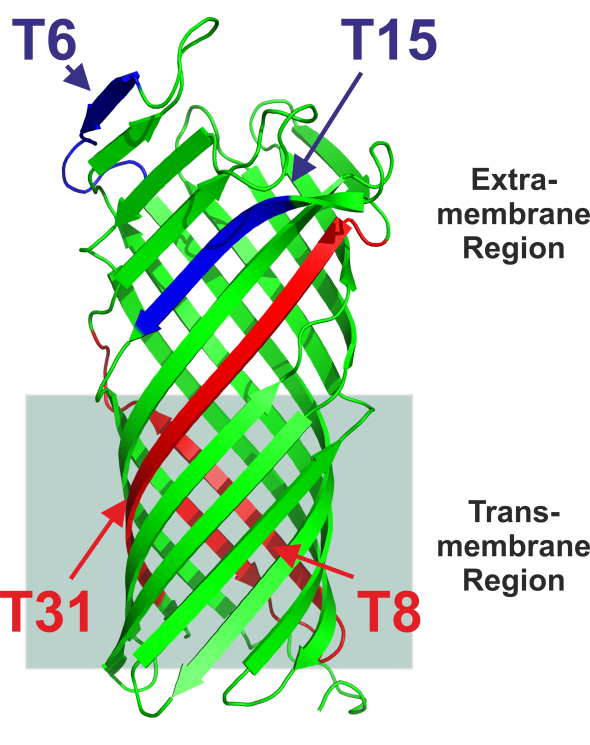


Supplementary Figure 3. Alternative representation of varying degrees of oxidation of different regions of OmpT in DDM detergent micelles or in A8-35 amphipol. Peptides that are modified to a greater extent in DDM or A8-35 are highlighted in blue and red, respectively. In the absence of knowing the specific modification sites, we see a greater extent of modification of the trans-membrane region in the amphipol A8-35, but a greater extent of modification of the extramembrane region in DDM detergent micelles.
